# Successful eradication of chronic myeloid leukemia in a child despite allogeneic graft rejection

**DOI:** 10.1002/cnr2.1663

**Published:** 2022-07-07

**Authors:** Susanna Vuorenoja, Kim Vettenranta, Olli Lohi

**Affiliations:** ^1^ Tampere Center for Child, Adolescent and Maternal Health Research, Tays Cancer Centre Tampere University Hospital, Tampere University Tampere Finland; ^2^ Pediatric Research Center, University of Helsinki Helsinki University Central Hospital, University of Helsinki Helsinki Finland

**Keywords:** CML, eradication, graft rejection, HSCT, reconstitution

## Abstract

**Background:**

Chronic myeloid leukemia (CML) is a rare disease in children and treated with tyrosine kinase inhibitors (TKI) and with allogeneic hematopoietic stem cell transplantation (HSCT) still in many cases.

**Case:**

We describe an 8‐year‐old patient with CML treated with two different TKIs before proceeding to allogeneic HSCT. Despite successful engraftment, prompt rejection of the graft was followed by autologous reconstitution. TKI therapy was reintroduced post‐rejection in anticipation of relapse but shortly discontinued due to low white blood cell and neutrophil counts. The patient has remained disease‐free over 5 years after graft rejection and without further therapy.

**Conclusion:**

This case suggests that even a short antileukemic effect by an allogeneic transplant may succeed in eradicating CML.

## 1 INTRODUCTION

Chronic myeloid leukemia (CML) is a myeloproliferative neoplasm rare in children and accounts for only 2%–3% of pediatric leukemias among the 1‐15‐year‐old and 9% of adolescents aged 15–19 years.^1^ CML is characterized by the presence of the Philadelphia chromosome (Ph+) leading to the generation of the Bcr‐Abl1 fusion gene. Allogeneic hematopoietic stem cell transplantation (HSCT) has traditionally been regarded as a treatment of choice for CML.^2^ Since 1990s, targeted therapy with tyrosine kinase inhibitors (TKI) has become the mainstay of treatment and around half of the adult patients can discontinue TKI without a relapse.^3^, ^4^, ^5^, ^6^ Likewise, in children with a sustained, deep, molecular remission (DMR) TKI therapy can be continued and HSCT avoided.^7^, ^8^

Here, we describe a child with CML who rapidly rejected the allogeneic graft but has remained disease‐free for over 5 years without a second transplant or further TKI therapy.

## 2 CASE

An 8‐year‐old patient was referred to a tertiary hospital (Tampere University Hospital, Finland) due to pain in the abdomen and limbs. The laboratory results showed an elevated white blood cell count (WBC) of 207 × 10E9/L(normal range 4.5–13.5 × 10E9/L) with a predominance of myelocytes in the blood smear, thrombocytosis of up to 716 × 10E9/L (normal range 180–400 × 10E9/L), and low hemoglobin of 87 g/L (normal range 110–155 × g/L). Bone marrow aspiration displayed expansion of the myeloid compartment. Fluorescence in situ hybridization and molecular genetics analyses revealed the presence of the major form (p210) of the Bcr‐Abl1 fusion gene in 98% of the cells, confirming the diagnosis of a chronic phase CML. Except for the enlarged spleen in abdominal ultrasound examination, no additional, aberrant clinical findings were observed.

**FIGURE 1:**
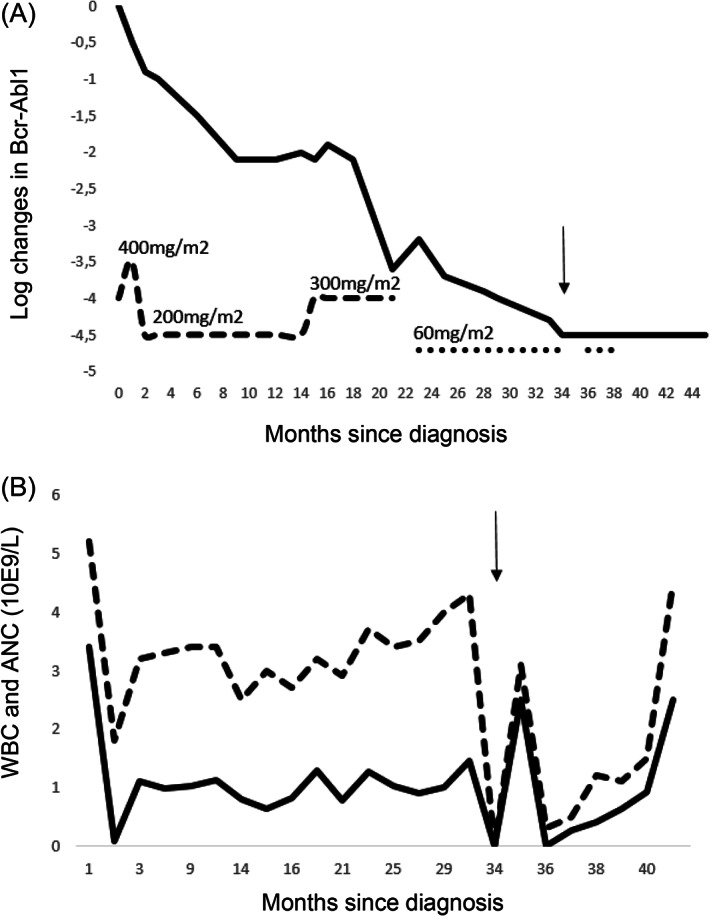
(A) The logarithmic change in peripheral blood *Bcr‐Abl1* transcript level as measured by RT‐PCR during treatment. The dashed line indicates the use of imatinib with different dosages, and the dotted line indicates the use of dasatinib. Timeline is shown in months, and the arrow indicates the timing of allogeneic HSCT (34 months from diagnosis). (B) Absolute neutrophil (ANC) and white blood cell (WBC) counts after initiation of imatinib therapy. There was a significant drop in the counts after 2 months of imatinib treatment and, therefore, imatinib was discontinued and later resumed at a reduced dose. HSCT was performed after 34 months of start of imatinib treatment. Two months after the HSCT, ANC dropped significantly and at 3 months post‐transplantation, the graft was rejected. Timeline is shown in months, and the arrow indicates the timing of allogeneic HSCT (34 months from diagnosis).

After a short pretreatment with hydroxyurea 1000 mg once a day, imatinib was commenced at a dose of 300 mg/m^2^. The dose was increased to 400 mg/m^2^ but shortly lowered to 200 mg/m^2^ to accommodate for a persisting neutropenia and pain in the legs, fingers, and wrists. The dose was, again, increased to 300 mg/m^2^ at 14 months of therapy. The patient achieved a complete hematologic response at 3 and complete cytogenetic response at 9 months after the initiation of treatment. Measurable residual disease (MRD) in peripheral blood as measured from Bcr‐Abl1 transcript by RT‐PCR, plateaued at the molecular response level 2 (MR2), and with persisting leg pain and fatigue, imatinib was substituted with dasatinib 60 mg/m^2^ at 21 months. Eight months later and 29 months since the introduction of TKI, a DMR (MR4) was achieved (Figure 1).

As allogeneic HSCT was considered the only curative therapy for CML at the time, 5 months after achieving MR4, HSCT from a 9/10 HLA‐matched, unrelated, female donor was performed at MR 4.3 in the peripheral blood. The patient was conditioned using fludarabine and thiotepa according to the institutional protocol, and dasatinib stopped before the initiation of the conditioning therapy. There were no early adverse events post‐transplant. On Day 21 post‐HSCT, the patient no longer needed red blood cell or platelet transfusions, and the neutrophil count reached 1 × 10E9/L on Day 32. Due to clinical signs of an engraftment syndrome, corticosteroids were initiated on Day 36. A routine GVHD prophylaxis was administered with daily cyclosporin together with methotrexate 10 mg/m^2^ twice a week for 2 weeks.

Two months post‐transplantation, the patient developed cytopenia and began needing weekly blood transfusions with a poor response. As an immunological mechanism was suspected, two doses of rituximab (375 mg/m^2^) were administered. However, at 3 months post‐transplantation, the BM sampling indicated hypoplasia and rejection of the graft with only 2% of donor cells persisting. MRD monitoring by Bcr‐Abl1 fusion PCR was negative. Pre‐emptive dasatinib at 60 mg/m^2^ was commenced in anticipation of a relapse but soon permanently discontinued due to low WBC and neutrophil counts (Figure 1B). Surprisingly, no evidence of disease has been detected by regular MRD monitoring in the peripheral blood (Figure 1A) or bone marrow. The patient has remained disease‐free for over 5 years since graft rejection without the reintroduction of TKI therapy. She is currently post‐pubertal with a height of +0.2 SD and weight at 10th percentile of the normal weight distribution.

## 3 DISCUSSION

We report here a child with CML who received an allogeneic HSCT but rejected the graft and spontaneously reconstituted her autologous, non‐leukemic hematopoiesis. The patient has remained disease‐free now for over 5 years without additional interventions, suggestive of a permanent eradication of the malignant CML clone.

Many pediatric patients with CML achieve an MMR or DMR with the TKI therapy, and the current recommendation is that patients in a sustained DMR continue with the TKI.^7^, ^8^, ^9^ Our patient had a suboptimal early response but did achieve a DMR after the introduction of a second‐generation TKI. She then proceeded to an allogeneic HSCT, the widely accepted curative treatment of choice for CML at the time. The recent approval and efficacy of the second‐generation TKIs, dasatinib and nilotinib, for the pediatric indication has further limited the role of HSCT, currently reserved mainly for patients with a poor response or intolerance to TKI therapy.^9^ In adults, treatment with TKI alone has resulted in life‐expectancies comparable to the age‐matched healthy population.^10^

Discontinuation of the TKI is an increasingly common practice in adults.^4^ In contrast, among children only limited data are available and mainly on patients who either stopped the TKI early due to poor compliance or otherwise discontinued successfully.^11^, ^12^ It remains unsettled whether TKI therapy is sufficient for a long‐term eradication of the CML in children as leukemia stem cells are not anticipated to be killed by the TKI alone.^13^, ^14^, ^15^, ^16^

The use of TKI therapy is accompanied with a specific set of toxicities in children. They may impair the growth and fertility and disrupt bone metabolism, thyroid function, and pubertal development.^17^, ^18^ To minimize the adverse effects, recent data on adults and a small series in children have suggested that patients in a sustained DMR can use an intermittent dosing schedule or discontinue the drug entirely.^12^, ^19^ Our patient experienced typical side‐effects of imatinib such as bone and joint pain and low WBC counts. Substitution of imatinib with dasatinib mitigated these symptoms and despite almost 3 years of TKI use, our patient has grown normally and underwent normal pubertal development.

It is well recognized that CML cells are sensitive to the graft‐versus‐leukemia effect rendered by an allogeneic graft, thereby justifying the use of HSCT as a treatment.^15^, ^20^ Prior to the TKI era, attempts were made to use autologous SCT for treatment of CML by using cells collected during remission and/or after purging of the graft.^21^, ^22^ This approach demonstrated that hematopoiesis can be reconstituted from patient's Ph‐negative cells. This is in agreement with our case, where the administration of high‐dose conditioning chemotherapy was followed by graft rejection and spontaneous reconstitution of the autologous non‐leukemic hematopoiesis.

Autologous hematopoietic reconstitution after allogeneic HSCT for CML has been described in adults both in the related and unrelated donor settings.^23^, ^24^ However, all reported cases have relapsed except for one case, where only 2‐year follow‐up was reported.^25^ Our case is the first child published with a successful, non‐leukemic, autologous recovery after the rejection of the allogeneic graft, and the first with a sustained remission and likely cure without re‐transplantation or long‐term use of TKI post‐transplantation.

An argument can be made on the patient not needing a transplant in the first place as she was in DMR at the time of transplantation. However, at the time of decision, an allogeneic HSCT was routine in aiming at the curative treatment of CML. It is also worth noting that her DMR had lasted no longer than 5 months before the transplantation and the first negative MRD came 3 months after transplantation. Second, we cannot entirely rule out a role for the conditioning chemotherapy in cure. With these caveats in mind, the case highlights the potent antileukemic effect of an allogeneic transplant in CML: even a short presence of immunologically active donor cells can elicit a robust immunological response to permanently eradicate the malignant CML clone.

## AUTHOR CONTRIBUTIONS

**Susanna Vuorenoja:** Conceptualization (equal); data curation (equal); writing – original draft (equal). **Kim Vettenranta:** Conceptualization (equal); writing – review and editing (equal). **Olli Lohi:** Conceptualization (equal); data curation (equal); writing – review and editing (equal).

## CONFLICT OF INTEREST

The authors declare no conflict of interest.

## ETHICS STATEMENT

The study was accepted by the Director of Science Center in Tampere University Hospital and informed consent was obtained from the patient and guardians before initiation of the study.

## Data Availability

The data that support the findings of this study are available from the corresponding author upon reasonable request.
